# Diversity in people's reluctance to use medical artificial intelligence: Identifying subgroups through latent profile analysis

**DOI:** 10.3389/frai.2022.1006173

**Published:** 2022-10-06

**Authors:** Haixia Wang, Qiaoqiao Sun, Li Gu, Kaisheng Lai, Lingnan He

**Affiliations:** ^1^School of Journalism and Communication, Jinan University, Guangzhou, China; ^2^Guangdong Medical Doctor Association, Guangzhou, China; ^3^School of Innovation Design, Guangzhou Academy of Fine Arts, Guangzhou, China; ^4^School of Communication and Design, Sun Yat-sen University, Guangzhou, China

**Keywords:** medical AI, objective knowledge, subjective knowledge, negative attitude, behavioral intention

## Abstract

Medical artificial intelligence (AI) is important for future health care systems. Research on medical AI has examined people's reluctance to use medical AI from the knowledge, attitude, and behavioral levels in isolation using a variable-centered approach while overlooking the possibility that there are subpopulations of people who may differ in their combined level of knowledge, attitude and behavior. To address this gap in the literature, we adopt a person-centered approach employing latent profile analysis to consider people's medical AI objective knowledge, subjective knowledge, negative attitudes and behavioral intentions. Across two studies, we identified three distinct medical AI profiles that systemically varied according to people's trust in and perceived risk imposed by medical AI. Our results revealed new insights into the nature of people's reluctance to use medical AI and how individuals with different profiles may characteristically have distinct knowledge, attitudes and behaviors regarding medical AI.

## Introduction

Medical artificial intelligence (AI) is critical to the future of medical diagnosis and can provide expert-level medical decisions. For example, in telemedicine, it is crucial to apply medical AI for diagnoses such as COVID-19 and skin cancer (Esteva et al., 2017; Hao, 2020; Hollander and Carr, 2020; Wosik et al., 2020). This advantage is particularly critical for improving the level of medical care in poor areas of developing countries (Topol, 2019). Despite this importance, there are many barriers to applying medical AI in health-care systems (Dwivedi et al., 2021). A multitude of studies have documented these barriers, including the public not having enough AI knowledge and people expressing negative attitudes toward medical AI in social media (Promberger and Baron, 2006; Eastwood et al., 2012; Price, 2018; Cadario et al., 2021). At the behavioral level, health-care system providers are reluctant to use medical AI, and patients hold doubts about using medical AI (Longoni et al., 2019). In light of previous studies on medical AI, it is critical for scholars to develop a better holistic understanding of how knowledge, negative attitudes and behavior factors are combined to influence the acceptance of medical AI by the population.

Thus far, the most common method to explore the obstacles in the application of medical AI is to ask people to self-report variables regarding their knowledge, attitude, and behavior toward AI and then to explore the relationships among these variables by using regression-based statistical analyses (Xu and Yu, 2019; Abdullah and Fakieh, 2020; Cadario et al., 2021). This approach represents a variable-centered method in which the unique relationships of each factor with other variables are explored (Marsh et al., 2009). However, such an approach does not reveal the ways in which individuals may have knowledge, negative attitudes and behavior factors that combine to shape their profile (Ekehammar and Akrami, 2003). For example, some individuals may have high knowledge while still having high negative attitudes toward medical AI. These ideas suggest that distinct profiles of medical AI likely exist. To investigate such a possibility, a person-centered approach is needed to explore the presence of distinct subpopulations of medical AI that differentially combine knowledge, negative attitudes and behavior (Zyphur, 2009; Wang and Hanges, 2011). Unfortunately, this approach to medical AI has mostly been overlooked. A person-centered approach allows researchers to understand how knowledge of and negative attitudes and behaviors toward medical AI conjointly shape profiles by capturing unobserved heterogeneity in the way people report their knowledge, negative attitudes and behaviors toward medical AI. These profiles can be leveraged to understand the barriers and further aid the application of medical AI. For example, the profile of low knowledge of but high negative attitude toward medical AI might be used to identify public policy to reduce the negative attitude toward medical AI by increasing the science knowledge of medical AI. Overall, there is value in examining whether there exist different profiles of barriers to medical AI.

To address these questions, we adopt the knowledge, attitudes and behavior (KAB) model (Kemm and Close, 1995; Yi and Hohashi, 2018) to understand the barriers to medical AI. The KAB model is particularly helpful and relevant for understanding and explaining the barriers to adopting medical AI. The core tenet of this model is that knowledge, attitudes, and behaviors are three related factors that are used to promote technology diffusion (Hohashi and Honda, 2015). Importantly, this model recognizes that these three factors are useful at identifying barriers to technology. Moreover, scholars have identified that the distinction between subjective knowledge and objective knowledge is important to understanding the barriers to medical AI. For instance, one recent study found that subjective knowledge of medical AI drives healthcare provider utilization (Cadario et al., 2021). Moreover, they found that greater subjective knowledge of medical decisions made by humans than medical AI providers contributes to medical AI aversion. Their findings imply how reluctance to utilize medical AI is driven both by the difficulty of subjectively understanding how medical AI makes decisions and by their objective understanding of human decision making. Drawing upon the KAB model, we investigate the profiles of heterogeneity in medical AI's objective knowledge, subjective knowledge, negative attitudes, and behavior.

Therefore, the objective of this research was to identify and describe the diversity in people's reluctance to use medical AI and its associated antecedents by employing latent profile analysis (LPA) (Woo et al., 2018). Specifically, we first tried to establish KAB profiles of medical AI in Study 1. Then, we sought to replicate and theoretically develop KAB profiles of medical AI in Study 2. Moreover, we tried to theoretically develop the KAB profiles by addressing the antecedents.

## Study 1: Establishing KAB profiles of medical AI

In Study 1, we use an inductive approach to establish profiles of medical AI (Woo and Allen, 2014). A person-centered approach can establish quantitatively distinct profiles that differ in the levels of objectivity and knowledge of and negative attitudes and behaviors toward medical AI; it can also create qualitatively distinct profiles varying in the relative degree of objective knowledge and subjective knowledge of negative attitudes and behaviors toward medical AI. For instance, one profile may include people with high objective and subjective knowledge of as well as negative attitudes and behavior toward medical AI, while another includes low levels of objective and subjective knowledge of as well as negative attitudes and behavior toward medical AI. Given the various combinations that may occur, we pose the following question:

*Research question: Are there distinct profiles of objective and subjective knowledge of and negative attitudes toward and behavior toward medical AI?*

## Study 1: Methods

### Participants and procedure

We recruited 328 participants online using convenience sampling. No participants were excluded. The participants provided informed consent and completed the survey. Table 1 provides demographic information on our sample.

**Table 1:** Demographic characteristics (*N* = 328).

**Variables**	**Frequencies (percentages)**
**Age**	
Mean (SD)	29.77 (8.18)
**Sex**	
Male	125 (38.1%)
Female	203 (61.9%)
**Education year**	
Mean (SD)	15.61 (1.75)
**Occupation**	
Full-time student	45 (13.7%)
Production	18 (5.5%)
Sales	23 (7.0%)
Public relations	19 (5.8%)
Customer service	11 (3.4%)
Administration	28 (8.5%)
Human resources	12 (3.7%)
Finance	21 (6.4%)
Clerical work	13 (4.0%)
Research	40 (12.2%)
Management	30 (9.1%)
Teaching	21 (6.4%)
Consulting	6 (1.8%)
Professional services (e.g., journalism and law)	22 (6.7%)
Other	19 (5.8%)

### Measures

#### Objective knowledge of medical AI

We used the Cadario et al. (2021) three-item multiple choice test to measure the participants' objective understanding of medical AI. Each item had one correct answer for medical AI. We scored objective knowledge of medical AI by summing the correct answers. Thus, the objective knowledge of medical AI ranged from 0 to 3 (*m* = 1.12, *SD* = 0.83). Before the formal measurement, we interviewed doctors to ensure the accuracy of objective knowledge and expert validity.

#### Subjective knowledge of medical AI

We used the Cadario et al. (2021) three-item scale to measure the participants' subjective knowledge of medical AI. The participants were asked to indicate the extent to which they agreed with the included statements (1 = “don't quite understand,” 5 = “quite understand”). One sample item is “To what extent do you feel that you understand what a medical AI algorithm considers when making the medical decision” (α = 0.74).

#### Negative attitudes of medical AI

We measured negative attitudes toward medical AI using an 8-item scale (Schepman and Rodway, 2020). The participants were asked to indicate their level of agreement with a list of statements (1 = “Strongly disagree,” 5 = “Strongly agree”). A sample item is as follows: “I find medical Artificial Intelligence sinister” (α = 0.84).

#### Behavioral intention of medical AI use

We measured the behavioral intention of medical AI use using a 5-item scale (Esmaeilzadeh, 2020). The participants were asked to indicate their level of agreement with a list of statements (1 = “Strongly disagree,” 5 = “Strongly agree”). A sample item is as follows: “I would like to use medical AI-based devices to manage my healthcare” (α = 0.84).

### Analytical approach

We first transformed raw measures of objective knowledge of medical AI, subjective knowledge of medical AI, negative attitudes toward medical AI, and behavioral intention toward medical AI use into z scores. Then, LPA was used to establish profiles of medical AI (Woo and Allen, 2014) using Mplus 8.3. We first established two profiles and then gradually increased the profiles until the model fitting index no longer improved (Nylund et al., 2007). For the model fitting index, referring to previous studies (Lo, 2001; Gabriel et al., 2015), we used the following: the log likelihood (LL), the free parameter (FP), the Akaike information criterion (AIC), the Bayesian information criterion (BIC), the sample-size-adjusted BIC (SSA–BIC), entropy, the bootstrap likelihood ratio test (BLRT), and the Lo-Mendell-Rubin likelihood ratio test (LMR). We consider the theoretical significance of the model and model indicators to identify the best-fitting model (Foti et al., 2012). The number of retained profiles should consider both the theoretical meaning of medical AI subpopulations and model indicators [lower LL, AIC, BIC, and SSA–BIC; higher entropy; and significant LMR (*p* < 0.05)].

## Study 1: Results

### Identification of profiles

**Figure 1:**
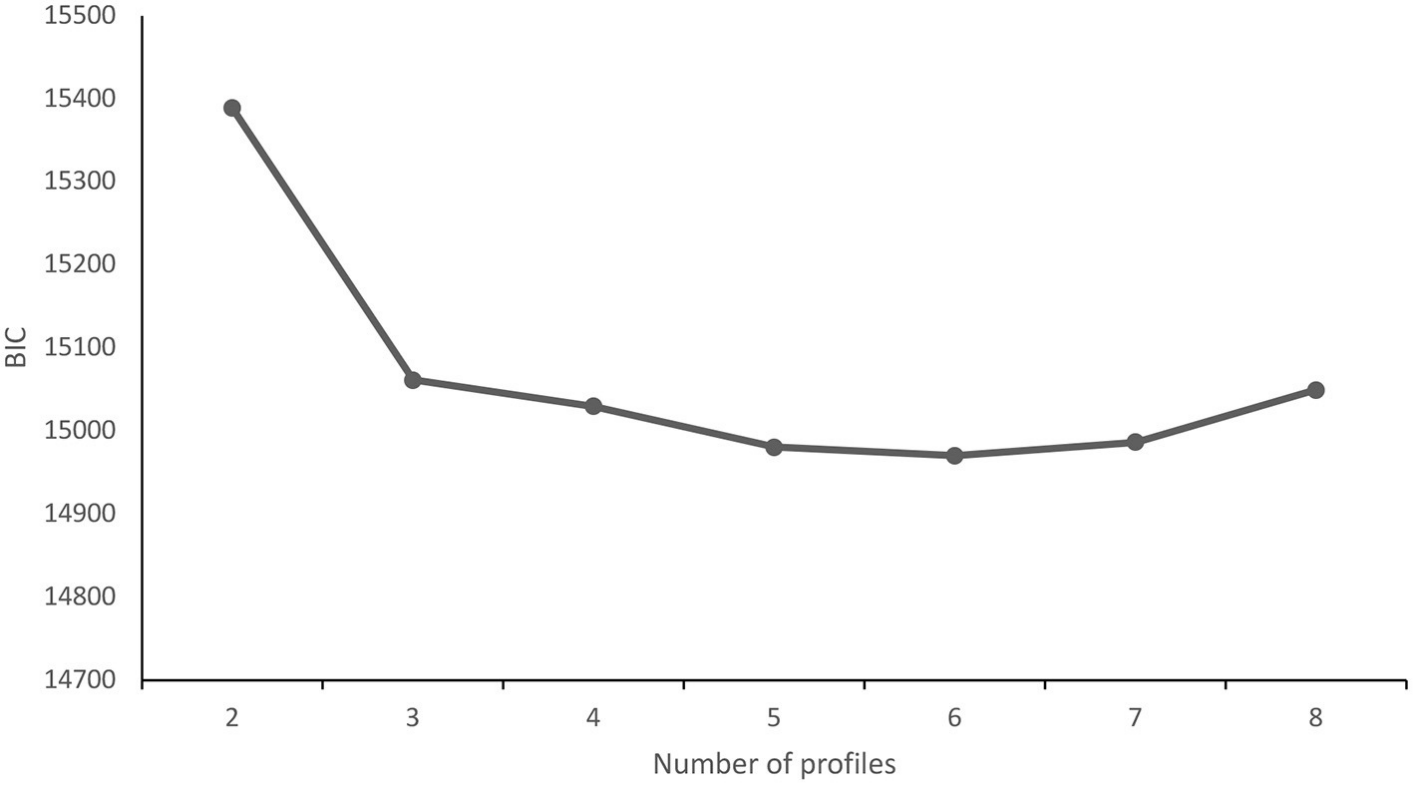
Goodness of fit of the BIC. The y-axis represents BIC (Bayesian information criterion); the x-axis represents the number of profiles (starting from 2).

Table 2 provides descriptive information for the study variables. As shown in Table 3, the 3-profile solution had low LL, AIC, and SSA-BIC. In addition, the elbow plot of BIC (Figure 1) shows that the slope of the BIC curve flattens around three profiles. Moreover, the 3-profile had significant LMR, unlike other solutions that had lower LL, AIC, and SSA-BIC. More importantly, the 3-profile had theoretical meaning for medical AI. Theoretically, as the number of profiles increased, these solutions contained redundant profiles of medical AI that modeled variants of the three main profiles. Thus, the 3-profile model can ensure theoretical parsimony while also meeting the statistical criterion. Together, these theoretical, visual and statistical considerations suggest that the 3-profile model is the best model with our data.

**Table 2:** Means, standard deviations, and correlations of study 1 variables (*N* = 328).

	***M*** **(*SD*)**	**1**	**2**	**3**	**4**	**5**	**6**
1. Age	29.77 (8.18)	–					
2. Sex	1.62 (0.49)	−0.21^**^					
3. Education years	15.61 (1.75)	−0.06	−0.01				
4. Negative attitudes	2.34 (0.71)	0.06	−0.05	0.01			
5. Objective knowledge	1.12 (0.83)	−0.08	−0.01	0.07	0.17^**^		
6. Subjective knowledge	3.40 (0.74)	0.06	−0.07	0.04	0.03	0.05	
7. Behavioral intentions	3.72 (0.61)	0.07	−0.07	0.03	−0.16^**^	0.01	0.45^**^

Sex (1 = male; 2 = female).

** p < 0.01.

**Table 3:** Fit statistics for profile solutions in study 1 and study 2.

**Number of profiles**	**LL**	**FP**	**AIC**	**BIC**	**SSA-BIC**	**Entropy**	**BLRT (*p*)**	**LMR (*p*)**
**Study 1 (*****N*** **= 328)**								
2	−7,543.868	52	15,191.735	15,388.972	15,224.029	0.917	0.0000	0.0368
3	−7,328.101	70	14,796.202	15,061.713	14,839.675	0.884	0.0000	0.0214
4	−7,259.976	88	14,695.953	15,029.738	14,750.604	0.878	0.0000	0.6511
5	−7,183.407	106	14,578.814	14,980.873	14,644.644	0.855	0.0000	0.3155
6	−7,126.079	124	14,500.159	14,970.492	14,577.168	0.876	0.0000	0.2367
7	−7,081.915	142	14,447.831	14,986.439	14,536.018	0.884	0.0000	0.8302
8	−7,061.320	160	14,442.640	15,049.522	14,542.006	0.892	0.2353	0.3077
**Study 2 (*****N*** **= 388)**								
2	−8,767.753	52	17,639.506	17,845.479	17,680.487	0.923	0.0000	0.0002
3	−8,573.748	70	17,287.497	17,564.767	17,342.663	0.927	0.0000	0.1050
4	−8,463.695	88	17,103.389	17,451.958	17,172.741	0.858	0.0000	0.4288
5	−8,367.262	106	16,946.523	17,366.390	17,030.061	0.858	0.0000	0.2004
6	−8,295.602	124	16,839.204	17,330.369	16,936.928	0.872	0.0000	0.5922
7	−8,229.962	142	16,743.925	17,306.387	16,855.834	0.889	0.0000	0.7110
8	−8,189.911	160	16,699.823	17,333.583	16,825.917	0.899	0.0000	0.6499

**Figure 2:**
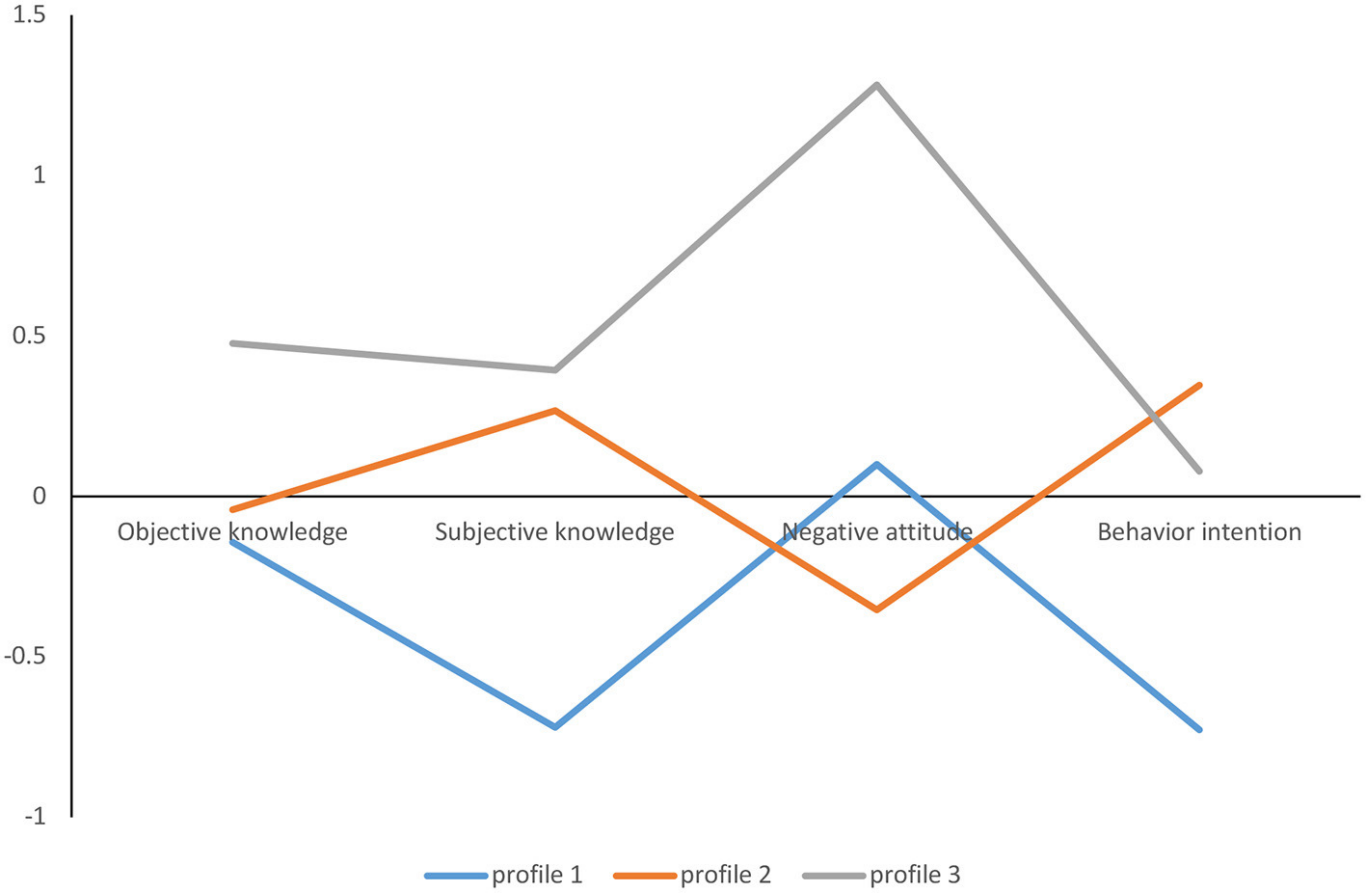
Latent profiles KAB profiles of medical AI. The y-axis refers to the mean Z score of the participants' objective knowledge, subjective knowledge, negative attitude, and behavioral intentions.

Table 4 shows descriptive information of the retained profiles. As shown in Figure 2, among the 328 people who completed the questionnaires, 92 (28%) participants were classified into subtype 1, which had the lowest objective and subjective knowledge of medical AI, yet they also had a middle level of negative attitudes toward medical AI and the lowest level of behavioral intention regarding medical AI use.

**Table 4:** Descriptive information of the retained profiles in study 1 and study 2.

**Profiles**	**% of sample**	**Objective knowledge**	**Subjective knowledge**	**Negative attitude**	**Behavior intention**
**Study 1 (*****N*** **= 328)**		***M*** **(*****SD*****)**	***M*** **(*****SD*****)**	***M*** **(*****SD*****)**	***M*** **(*****SD*****)**
1	28%	0.97 (0.76)	2.72 (0.65)	2.45 (0.47)	3.05 (0.53)
2	58%	1.10 (0.83)	3.65 (0.59)	1.98 (0.42)	4.03 (0.36)
3	14%	1.51 (0.84)	3.73 (0.61)	3.66 (0.45)	3.80 (0.51)
**Study 2 (*****N*** **= 388)**					
1	24%	1.42 (0.88)	2.99 (0.67)	2.63 (0.44)	3.13 (0.61)
2	68%	1.45 (0.76)	3.64 (0.67)	1.84 (0.38)	4.15 (0.38)
3	8%	1.58 (0.85)	3.56 (0.65)	3.76 (0.40)	3.63 (0.62)

A total of 191 (58%) participants were classified as subtype 2. The participants in this subtype showed a moderate level of objective and subjective knowledge of medical AI, yet they had the lowest negative attitudes toward medical AI and the highest behavioral intention toward medical AI use.

Forty-five (14%) participants were classified as subtype 3. The participants in this subtype showed a high level of objective and subjective knowledge of medical AI, yet they had the highest level of negative attitudes toward medical AI and a middle level of behavioral intention toward medical AI use.

## Study 2: Replication and theoretical development of KAB profiles of medical AI

We first intended to replicate the main results of Study 1; thus, we expected to find the same 3 profiles of medical AI. Accordingly, we seek to explore the following question:

*Research question 1: Will three distinct KAB profiles of medical AI emerge?*

We also expected to extend Study 1 by examining the antecedents of KAB profiles of medical AI in Study 2. When exploring the KAB profiles, it is critical to identify factors that can predict KAB profile membership. Previous research argues that individuals' trust and risk perception of medical AI predict their reluctance to use medical AI (Esmaeilzadeh, 2020). Thus, we pose the following question:

*Research question 2: Do trust perception of medical AI and risk perception of medical AI predict KAB profile membership?*

### Participants and procedure

We recruited 388 participants. No participants were excluded. The participants provided informed consent and completed the survey. Table 5 provides demographic information on our sample.

**Table 5:** Demographic characteristics (*N* = 388).

**Variables**	**Frequencies (percentages)**
**Age**	
Mean (SD)	30.48 (8.47)
**Sex**	
Male	158 (40.7%)
Female	230 (59.3%)
**Education year**	
Mean (SD)	15.82 (1.67)
**Occupation**	
Full-time student	47 (12.1%)
Production	14 (3.6%)
Sales	30 (7.7%)
Public relations	15 (3.9%)
Customer service	7 (1.8%)
Administration	36 (9.3%)
Human resources	12 (3.1%)
Finance	36 (9.3%)
Clerical work	21 (5.4%)
Research	68 (17.5%)
Management	40 (10.3%)
Teaching	17 (4.4%)
Consulting	0 (0%)
Professional services (e.g., journalism, law)	31 (8.0%)
Other	14 (3.6%)

### Measures

#### Objective knowledge of medical AI

We used the same three-item multiple choice test to measure the participants' objective understanding of medical AI as in Study 1.

#### Subjective knowledge of medical AI

We used the same three items to measure subjective knowledge of medical AI as in Study 1 (α = 0.75).

#### Negative attitudes of medical AI

We used the same 8 items to measure negative attitudes toward medical AI as in Study 1 (α = 0.85).

#### Behavioral intention of medical AI use

We used the same 5 items to measure behavioral intention regarding medical AI use as in Study 1 (α = 0.79).

#### Trust perception of medical AI

We measured the behavioral intention toward medical AI use using a 5-item scale (Esmaeilzadeh, 2020). The participants were asked to indicate their level of agreement with the statements (1 = “Strongly disagree,” 5 = “Strongly agree”). A sample item is as follows: “I trust the medical AI algorithms used in healthcare” (α = 0.77).

#### Risk perception of medical AI

We measured the behavioral intention toward medical AI use using a 5-item scale (Esmaeilzadeh, 2020). The participants were asked to indicate their level of agreement with the statements (1 = “Strongly disagree,” 5 = “Strongly agree”). A sample item is as follows: “The risk of using medical AI-based tools for medical purposes is high” (α = 0.85).

## Study 2: Results

### Replicating profiles

**Figure 3:**
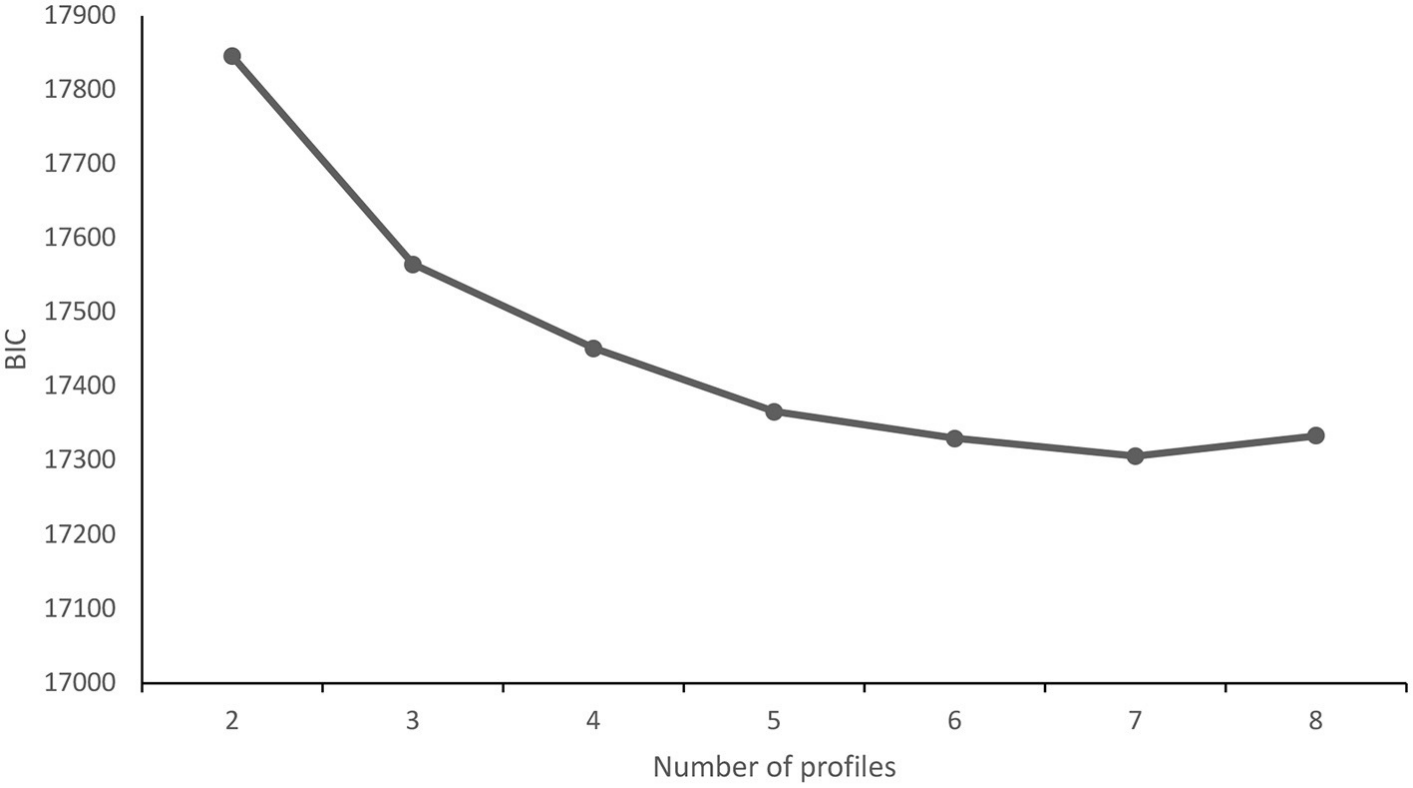
Goodness of fit of the BIC. The y-axis represents BIC (Bayesian information criterion); the x-axis represents the number of profiles (starting from 2).

Table 6 reports descriptive information for our variables. Table 3 reports fit information for profile solutions. Table 4 illustrates descriptive information for the retained three-profile solution. The three-solution was chosen because it had lower AIC, BIC, and SSA-BIC. It also had the highest entropy. Moreover, the elbow plot of BIC (Figure 3) shows that the slope of the curve flattens around three profiles. Theoretically, when the number of profiles of medical AI increased, these profile solutions contained redundant profiles that included variants of the three main medical AI profiles. Thus, to ensure theoretical parsimony, we identified the three-profile solution as the best-fitting model for our data.

**Table 6:** Means, standard deviations, and correlations of study 2 variables (*N* = 388).

	***M*** **(*SD*)**	**1**	**2**	**3**	**4**	**5**	**6**
1. Age	30.48 (8.47)	–					
2. Sex	1.59 (0.49)	−0.13^*^					
3. Education (years)	15.82 (1.67)	−0.20^**^	−0.07				
4. Negative attitudes	2.18 (0.70)	−0.03	0.02	−0.01			
5. Objective knowledge	1.45 (0.79)	−0.02	0.00	−0.01	0.06		
6. Subjective knowledge	3.48 (0.72)	0.05	0.08	0.10	−0.14^**^	−0.02	
7. Behavioral intentions	3.86 (0.64)	0.11^*^	−0.05	−0.05	−0.44^**^	−0.02	0.40^**^

Sex (1 = male; 2 = female).

* p < 0.05.

** p < 0.01.

**Figure 4:**
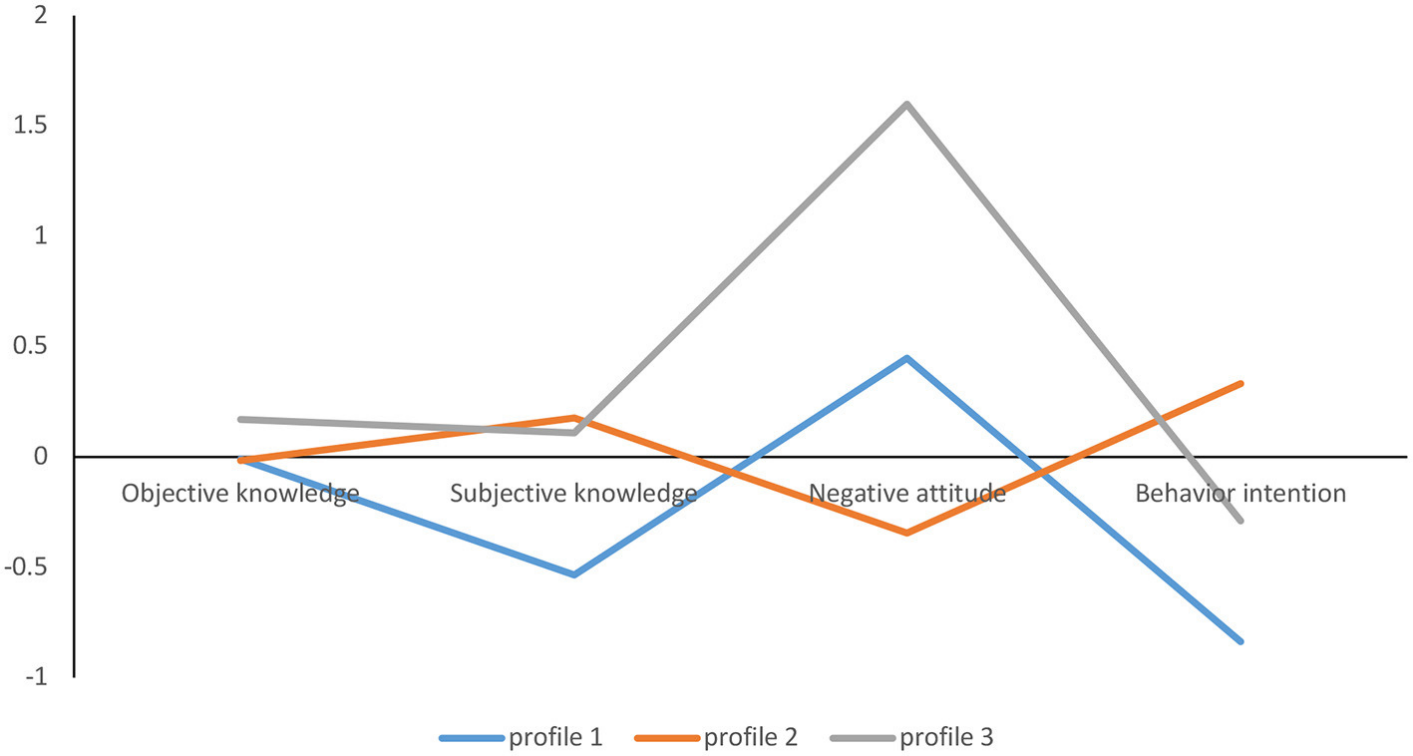
Latent profiles KAB profiles of medical AI. The y-axis refers to the mean Z score of the participants' objective knowledge, subjective knowledge, negative attitudes, and behavioral intentions.

For research question 1, we replicated the three profiles as in Study 1. As shown in Figure 4, among the 388 people who completed the questionnaires, 93 (24%) participants were classified into subtype 1, which had the lowest objective and subjective knowledge of medical AI, yet they also had a middle level of negative attitudes toward medical AI and the lowest level of behavioral intention toward medical AI use.

A total of 264 (68%) participants were classified into subtype 2. The participants in this subtype showed a moderate level of objective and subjective knowledge of medical AI, yet they had the lowest negative attitudes toward medical AI and the highest behavioral intention toward medical AI use.

Thirty-one (8%) participants were classified as subtype 3. The participants in this subtype showed a high level of objective and subjective knowledge of medical AI, yet they had the highest level of negative attitudes toward medical AI and a middle level of behavioral intention toward medical AI use.

### Examination of antecedents

Regarding antecedents, following previous studies (Vermunt, 2010; Asparouhov and Muthén, 2014), we used the RESTEP to test which variables are related to the profiles of medical AI. As shown in Table 7, we found that trust perception of medical AI, risk perception of medical AI, whether AI would replace my job, AI's benefit in medicine, and whether AI cooperates with humans are significant antecedents of the KAB profile membership of medical AI. Specifically, those perceiving a higher trust perception of medical AI were more likely to be in profile 2 [odds ratios (OR) = 14.91, *p* = 0.027] than in profile 1. Those perceiving a higher risk perception of medical AI were more likely to be in profiles 1 [odds ratios (OR) = 3.22, *p* = 0.026] and 3 (OR = 4.70, *p* = 0.048) than in profile 2. Those perceiving a higher perception of medical AI replacing my job were less likely to be in profile 3 [odds ratios (OR) = 0.17, *p* = 0.000] than in profile 2. Those perceiving a higher perception of medical AI's benefit in medicine were less likely to be in profiles 1 [odds ratios (OR) = 0.41, *p* = 0.000] and 3 (OR = 0.27, *p* = 0.000) than in profile 2. Those perceiving a higher trust perception of medical AI were more likely to be in profiles 2 [odds ratios (OR) = 14.91, *p* = 0.027] and 3 (OR = 2.84, *p* = 0.048) than in profile 1. Those perceiving a higher cooperation between medical AI and humans were less likely to be in profile 1 [odds ratios (OR) = 0.44, *p* = 0.00] than in profile 3. Those perceiving a higher cooperation between medical AI and humans were less likely to be in profile 1 [odds ratios (OR) = 0.54, *p* = 0.00] than in profile 2. We found no other significant results.

**Table 7:** Three-step results for antecedents (RESTEP) in study 2.

**Antecedents**	**Profile 1 v. 2**	**Profile 1 v. 3**	**Profile 2 v. 3**
Trust of medical AI	−2.702^***^	−1.045^*^	1.657^**^
Risk of medical AI	1.169^***^	−0.379	−1.548^***^
AI replacing job	−0.444	1.330^*^	1.774^***^
AI benefit in medicine	−0.885^**^	0.408	1.293^**^
AI cooperation with humans	−0.608^*^	−0.820^*^	−0.212

* p < 0.05.

** p < 0.01.

*** p < 0.001.

## Discussion

### Summary of the findings

The results of this study showed that there is heterogeneity in people's medical AI use. We identified 3 profiles based on objective knowledge and subjective knowledge of and negative attitudes and behavioral intentions toward medical AI. First, the participants in profile 1 had the lowest objective and subjective knowledge of medical AI, yet they also had a middle level of negative attitudes toward medical AI and the lowest level of behavioral intention regarding medical AI use. Second, the participants in profile 2 showed a moderate level of objective and subjective knowledge of medical AI, yet they had the lowest negative attitudes toward medical AI and the highest behavioral intention toward medical AI use. Third, the participants in profile 3 showed a high level of objective and subjective knowledge of medical AI, yet they had the highest level of negative attitudes toward medical AI and a middle level of behavioral intention toward medical AI use.

### Theoretical implications

Our research makes a variety of theoretical contributions. First, by taking a person-centered approach that categorized people into different profiles based upon their objective and subjective knowledge of and negative attitudes and behavioral intentions toward medical AI, our results depict a more holistic picture of people who are reluctant to use medical AI (Wang and Hanges, 2011). Most of our sampled individuals fell into profile 2, supporting the KAB model's hypothesis that knowledge, attitudes and behavior are related (Yi and Hohashi, 2018). That is, individuals with high knowledge have a low negative attitude and high behavioral intentions toward objects. The existence of profile 3 departs from the argument of the KAB model and the predominant variable-centered method that suggests links among knowledge, attitude and behavior instead highlighting the idea that these attributes and actions separately shape individuals' holistic picture of medical AI.

Second, while the KAB model (Chaffee and Roser, 1986; Abera, 2003) provides a useful lens through which to view the diversity of people's medical AI use, our study also gives back to this theory by revealing the shortcomings of this model. Notably, across the two samples, we did not observe a profile characterized by a middle level of objective and subjective knowledge of and middle levels of negative attitudes and behavioral intention toward medical AI, which could be a reasonable prediction derived from the KAB model. One potential explanation for this pertains to the complexity and heterogeneity of medical AI use (Cadario et al., 2021). That is, the barriers to medical AI use are not a simple phenomenon that can be completely explained by the KAB model. Instead, there is considerable heterogeneity in individuals reluctant to use medical AI. Thus, when considering complex phenomena such as medical AI use, we cannot simply use KAB to apply to this context and come to a simple conclusion.

Third, our work supports and extends the KAB model on the role of trust perception and risk perception in shaping individuals' medical AI use by developing and operationalizing a coherent framework of antecedents of medical AI use profiles. Consistent with the AI literature (Esmaeilzadeh, 2020; Dwivedi et al., 2021), trust and risk perception, AI replacing the jobs of humans, AI's benefit in medicine and AI's cooperation with humans were differentially related to medical AI use profile.

### Practical implications

Our study results provide many practical insights indicating the importance of helping individuals, media communicators, medical doctors and enterprise managers make sense of the complexity and heterogeneity of individuals' reluctance to use medical AI. For example, medical doctors could realize that some people exhibit consistent knowledge of and attitudes and behaviors toward medical AI, but others exhibit more variability in these domains, so there is no way to reach a simple and general conclusion about this subject. Importantly, our results highlight the importance of recognizing that there may be disassociation between someone's knowledge of and negative attitudes toward medical AI. Decision makers should be cautious when giving advice to individuals even if the individuals appear to have high knowledge of medical AI. Last, decision makers and policy makers may be able to create personalized intervention and dissemination programs to improve people's knowledge of AI, especially their subjective knowledge, and to help individuals in need actively adopt AI in seeking medical care in the future.

### Limitations and future directions

Our research has several limitations, which may offer fruitful directions for future research. First, future research may build upon our findings to explore whether the three identified profiles of medical AI exist and new profile(s) emerge in different cultural contexts with different samples to address the representativeness of the sample. Second, as people's knowledge of and negative attitudes and behavioral intentions toward medical AI might change over time, it is possible to employ latent transition analysis (Collins and Lanza, 2009) to address the shift in the KAB profile of medical AI. Third, in our study, objective knowledge and subjective knowledge were consistent, and there was no significant difference in shaping the profile of medical AI. This may be because our sample is the general public, and there is no significant difference between their objective and subjective knowledge of medical AI. However, for professionals, such as doctors, it is still worth exploring the effects of age in shaping people's reluctance to use medical AI in depth.

## Conclusion

The burgeoning AI literature has been limited in its understanding of the diversity in people's reluctance to use medical AI. We used LPA to better understand the heterogeneity of people's reluctance to use medical AI regarding their knowledge, negative attitudes and behavioral intentions. Our results demonstrated that different medical AI profiles consistently exist, and it is helpful to use a person-centered approach to better understand the complexity of obstacles in people's reluctance to use medical AI.

## Data availability statement

The raw data supporting the conclusions of this article will be made available by the authors, without undue reservation.

## Ethics statement

The studies involving human participants were reviewed and approved by the Ethical Committee of Jinan University. The patients/participants provided their written informed consent to participate in this study.

## Author contributions

HW, KL, and LH conceived and designed the research. HW and LH performed the research. HW, KL, QS, and LG analyzed the data and wrote the manuscript. All authors contributed to the article and approved the submitted version.

## Funding

This work was supported by the Program of National Natural Science Foundation of China (Grant Numbers 72174075 and 71801109) and Humanity and Social Science Youth Foundation of Ministry of Education of China (Grant Numbers 19YJCZH073).

## Conflict of interest

The authors declare that the research was conducted in the absence of any commercial or financial relationships that could be construed as a potential conflict of interest.

## Publisher's note

All claims expressed in this article are solely those of the authors and do not necessarily represent those of their affiliated organizations, or those of the publisher, the editors and the reviewers. Any product that may be evaluated in this article, or claim that may be made by its manufacturer, is not guaranteed or endorsed by the publisher.
