# Supplementary material for: Diversity in people's reluctance to use medical artificial intelligence: Identifying subgroups through latent profile analysis
Source: Front Artif Intell. 2022 Oct 6;5:1006173. doi: 10.3389/frai.2022.1006173 (PMC9583399; doi:10.3389/frai.2022.1006173)
Supplement: Supplementary file 1 [file Data_Sheet_1.docx]

Supplemental files

Scales used in our research

*Objective knowledge of medical AI*

1当通过医学影像图片判断疾病严重程度时，人工智能会怎么做？

A 考虑这个图片显示的疾病的单个特征，比如说逐一检查癌症影像图片的颜色、边界、对称性等

B 考虑这个图片与其他同类型的图片的相似性，比如说通过原始影像图片像素来比对与以往癌症影像图片的相似性

C 基于图片的核心特征来做判断，比如说通过癌症影像图片的形状来判断

2当通过医学影像图片判断疾病严重程度时，人工智能会怎么做？

A 考虑一系列诊断标准，并且按照一定的顺序一次诊断一个标准

B 同时考虑一系列诊断标准

C 模拟疾病发展模式

3 当通过医学影像图片做出诊断结果时，人工智能会怎么做？

A 计算疾病发生的概率：0%到100%

B 只做出低风险或高风险类别的诊断结果

C 做出有病或无病的结果

Reference

Cadario, R., Longoni, C., & Morewedge, C. K. (2021). Understanding, explaining, and utilizing medical artificial intelligence. *Nature Human Behaviour*, *5*(12), 1636–1642. https://doi.org/10.1038/s41562-021-01146-0.

*Subjective knowledge of medical AI*

1您在多大程度上了解人工智能进行医疗疾病诊断时的决策标准？

2您在多大程度上理解人工智能进行医疗疾病时的检查过程？

3您在多大程度上理解人工智能做出医疗疾病决策后的结果？

Reference

Cadario, R., Longoni, C., & Morewedge, C. K. (2021). Understanding, explaining, and utilizing medical artificial intelligence. *Nature Human Behaviour*, *5*(12), 1636–1642. https://doi.org/10.1038/s41562-021-01146-0.

*Negative attitudes of medical AI*

1医疗组织在不道德地使用人工智能

2我认为医疗人工智能会犯很多错误

3我觉得医疗人工智能是邪恶的

4医疗人工智能可能会控制人类

5我认为医疗人工智能是危险的

6当想到未来使用医疗人工智能时，我感到非常不安

7如果医疗人工智能被越来越多地使用，像我这样的人会遭殃

8医疗人工智能被用来监视人

Reference

Schepman, A., & Rodway, P. (2020). Initial validation of the general attitudes towards artificial intelligence scale. *Computers in Human Behavior Reports*, *1*, 100014. https://doi.org/10.1016/j.chbr.2020.100014.

*Behavioral intention of medical AI use*

1我同意在临床上使用人工智能

2我会考虑将人工智能用于医疗目的

3我想使用人工智能来管理我的医疗工作

4在未来，我愿意使用人工智能进行诊断和治疗

5我很可能会采纳人工智能提供的医疗规划建议

Reference

Esmaeilzadeh, P. (2020). Use of AI-based tools for healthcare purposes: A survey study from consumers’ perspectives. *BMC Medical Informatics and Decision Making*, *20*(1), 170. https://doi.org/10.1186/s12911-020-01191-1.
